# The miR-183/96/182 cluster is upregulated in glioblastoma carrying *EGFR* amplification

**DOI:** 10.1007/s11010-022-04435-y

**Published:** 2022-04-29

**Authors:** Björn Schneider, Doreen William, Nora Lamp, Annette Zimpfer, Christian Henker, Carl Friedrich Classen, Andreas Erbersdobler

**Affiliations:** 1 Institute of Pathology, University Medicine Rostock, Strempelstr. 14, 18057 Rostock, Germany; 2 Children and Adolescents Hospital, University Medicine Rostock, Ernst-Heydemann-Str. 8, 18057 Rostock, Germany; 3 Present Address: ERN-GENTURIS, Hereditary Cancer Syndrome Center Dresden, Institute for Clinical Genetics, University Hospital Carl Gustav Carus at the Technische Universität Dresden, Dresden, Germany; 4 Department of Neurosurgery, University Medicine Rostock, Schillingallee 35, 18057 Rostock, Germany

**Keywords:** Glioblastoma, *EGFR* amplification, microRNA deregulation, miR-183/96/182 cluster, FOXO1 expression

## Abstract

Glioblastoma (GBM) is one of the most frequent primary brain tumors. Limited therapeutic options and high recurrency rates lead to a dismal prognosis. One frequent, putative driver mutation is the genomic amplification of the oncogenic receptor tyrosine kinase *EGFR*. Often accompanied by variants like *EGFRvIII*, heterogenous expression and ligand independent signaling render this tumor subtype even more difficult to treat, as EGFR-directed therapeutics show only weak effects at best. So *EGFR*-amplified GBM is considered to have an even worse prognosis, and therefore, deeper understanding of molecular mechanisms and detection of potential targets for novel therapeutic strategies is urgently needed. In this study, we looked at the level of microRNAs (miRs), small non-coding RNAs frequently deregulated in cancer, both acting as oncogenes and tumor suppressors. Comparative analysis of GBM with and without *EGFR* amplification should give insight into the expression profiles of miRs, which are considered both as potential targets for directed therapies or as therapeutic reagents. Comparison of miR profiles of *EGFR*-amplified and *EGFR*-normal GBM revealed an upregulation of the miR-183/96/182 cluster, which is associated with oncogenic properties in several tumor entities. One prominent target of this miR cluster is FOXO1, a pro-apoptotic factor. By observing FOXO1 downregulation in *EGFR*-amplified tumors, we can see a significant correlation of *EGFR* amplification, miR-183/96/182 cluster upregulation, and repression of FOXO1. Although no significant difference in overall survival is shown, these data may contribute to the molecular understanding of this tumor subtype and offer potential targets for miR-based therapies.

**Supplementary Information:**

The online version contains supplementary material available at 10.1007/s11010-022-04435-y.

## Introduction

Glioblastoma (GBM) is one of the most frequent and fatal primary malignancy of the central nervous system. Despite advances in therapy, the prognosis remains dismal with a median survival of about 12–14.6 months after diagnosis and full treatment [1], which standardly consists of surgical resection, radiotherapy, and chemotherapy with temozolomide (TMZ) [1, 2]. However, the recurrence rate is very high and diffuse infiltrative growth makes complete tumor removal nearly impossible. Given the high inter- and intratumoral heterogeneity of GBM [3], increased efforts in molecular profiling are necessary to facilitate development of more targeted therapy options to overcome the limitations of the current standard GBM treatment. One frequent mutation is the amplification of the receptor tyrosine kinase *EGFR*, which occurs in almost 60% of primary GBM [4] and is associated with a worse prognosis [5, 6] Increased *EGFR* activity leads to enhanced signaling in several tumor associated downstream pathways like RAS or PI3K (reviewed in [7] and [8]). In around 50% of tumors carrying an *EGFR* amplification, accompanying expression of additional mutational variants is seen [8], the most frequent being *EGFRvIII*, a deletion variant lacking exons 2–7 leading to a loss of the extracellular ligand-binding domain. The ability to heterodimerize with wild-type EGFR and other receptors of the ERBB family leads to ligand independent signaling [9] and, therefore, to ectopic activation of downstream targets. Furthermore, the existence of different forms of aberrant EGFR leads to a heterogenous expression pattern and, therefore, heterogenous signaling abilities [10], making this tumor entity even more difficult to target with specific therapeutic approaches. Indeed, in contrast to several other tumor entities (lung, colon), targeting EGFR and/or EGFRvIII with inhibitors, or antibodies showed hardly any significant therapeutic response in GBM [7, 11–13]. Possible reasons for resistance to EGFR/EGFRvIII-targeted therapy are blood–brain barrier, tumor heterogeneity, extrachromosomal localization of *EGFR*, and *EGFRvIII* amplicons in double minutes, and mutation of genes in downstream pathways [7]. Furthermore, tyrosine kinase inhibitors are rather inefficient, as they specifically target sites of the kinase domain which are mutated for instance in lung cancer, but not in GBM [7]. EGFR-directed antibodies seem not to be able to inactivate all mutant forms like EGFRvIII, as they rather lead to protein recycling than to degradation [13]. As direct targeting of EGFR and EGFRvIII proved difficult and, so far, inefficient, other therapeutic strategies are urgently need. One potential level of targeted therapy approaches could be the level of microRNAs (miRs). MiRs are small non-coding RNAs, which bind to their target mRNAs to inhibit protein expression. They are frequently deregulated in multiple cancers. Deregulation can occur at any point of miR-biogenesis and has, depending on the miR and tissue or tumor type involved, oncogenic or tumor suppressive effects [14]. Importantly, a given miR can be oncogenic in one tumor type, and tumor suppressive in another [15]. In GBM, several miRs, like miR-21 with oncogenic or miR-34a with tumor suppressive properties, are involved in tumorigenesis, impacting diverse cancer signatures therein [16]. Furthermore, miRs are deemed therapeutic targets and can be inhibited or re-expressed in tumors [17]. Therefore, application of anti-miRs coupled to nanoparticles, shown to be functional in vitro, is hypothesized to successfully cross the blood–brain barrier to deliver the therapeutic agents directly to the tumor [18, 19]. Additionally, several miRs have been identified to repress MGMT expression and increase sensitivity to alkylating agents [20].

Information about correlations of *EGFR* alterations and changes of miR expression is scarce. One study showed a change of miR-9 expression in vitro for cells expressing EGFRvIII [9, 21]. Another study comparing miR profiles showed downregulation of miR-200c correlating with *EGFR* amplification [22]. To assess whether *EGFR* amplification has an influence on miR expression, we compared the miR expression profiles of *EGFR* amplified to those of *EGFR*-normal GBM. The aim of this study is the identification of commonly differentially expressed miRs and/or their target genes, which could give hints to potential targets for miR-associated or otherwise targeted therapeutic strategies. Our results show a significant upregulation of the miR-183/96/182 cluster in *EGFR*-amplified tumors. This cluster, consisting of miR-183, miR-96, and miR-182, is deregulated in different tumor entities and mostly considered as oncogenic [23]. Although members of the cluster are known to play a role in GBM [24, 25], no correlation of *EGFR* amplification and miR-183/96/182 cluster upregulation has been reported to date. Additionally, FOXO1, a prominent target of the miR-183/96/182 cluster [23] and associated with pro-apoptotic and tumor suppressive properties [26–28] is downregulated in *EGFR*-amplified GBM. Our findings should contribute to a better understanding of the role of microRNAs in *EGFR*-amplified GBM and help facilitate the development of new therapeutic strategies, such as those combining miR-based approaches with, so far widely inefficient, *EGFR* inhibition.

## Materials and methods

### Tumor samples

Formalin-fixed paraffin-embedded (FFPE) tumor material from glioblastoma patients was obtained from the archive of the Institute for Pathology, University Medicine, Rostock. Specimen collection was conducted in accordance with the ethics guidelines for the use of human material, approved by the Ethics Committee of the University of Rostock (Reference number: A 2009/34) and with informed written consent from all patients prior to surgery. Hematoxylin and eosin (H&E)-stained sections were examined by an experienced pathologist to ensure sample sufficiency and quality. Samples with high content of necrotic tissue were excluded from the study. Patient data are listed in Supplementary Table 1.

### Tissue microarrays

Tissue microarrays (TMA) were created using a Manual Tissue Arrayer MTA-1 (Beecher Instruments, Sun Prairie, WI, USA) with 1-mm diameter punches. From the donor blocks, three punches per sample were taken from tumor regions suitable for analysis (high tumor cell content, no necrosis) and transferred to an empty acceptor block. Afterwards, blocks were heated to 50 °C, and the correct placement of the punches was confirmed by microscopic examination. A final quality check of the TMA was done by H&E staining to confirm that all punches contained the desired amount of tumor tissue for further analysis.

### Chromogenic in situ hybridization

For determination of *EGFR* amplification status, two-colored chromogenic in situ hybridization (2C CISH) was performed. Microtome sections of 4 µm thickness of samples or TMA were mounted on coated slides. *EGFR*-specific 2C CISH was performed using the ZytoDot 2C CISH implementation Kit (Zytomed Systems, Berlin, Germany) and the ZytoDot 2C SPEC EGFR/CEN 7 Probe (Zytomed Systems) according to the manufacturer’s protocol. Analysis of the processed samples was performed with bright-field microscopy using a 40× objective. Each red signal specifically represented the centromere of chromosome 7 for reference and ploidy determination. Green signals were specific for the *EGFR* gene. Cells were considered to carry *EGFR* amplification if the ratio of green signals to red signals was greater than 2 or if green signals occurred in clusters.

### microRNA extraction

For microRNA extraction, the miRNeasy FFPE-Kit (Qiagen, Hilden, Germany) was used for FFPE sections and the Isolate II miRNA Kit (Bioline, Luckenwalde, Germany) for cell pellets, following the manufacturers’ protocols. Concentration of extracted RNA was determined with a Nanodrop spectrometer (Peqlab, Erlangen, Germany).

### miR screening arrays

Analysis of microRNA expression was performed using the Nanostring nCounter System with the Human v3 miRNA assay (Nanostring, Seattle, WA, USA). The analysis procedure was performed with 250 ng per sample by a service provider lab (Transcriptome and Genome Analysis Laboratory (TAL), Microarray and Deep-Sequencing Facility, University Medicine Göttingen) and analyzed by the authors with the nSolver 2.6 software (Nanostring) using standard settings for background subtraction and normalization. Group-wise comparison (*EGFR*-amp vs. *EGFR*-norm) was performed, delivering fold change and *p* values.

### microRNA-specific quantitative PCR

Expression analysis of miR-183-5p, miR-96-5p, and miR-182-5p was performed by qPCR using miR-specific TaqMan Assays (Applied Biosystems, Darmstadt, Germany), containing RT-primers specific for the mature miR and corresponding primer/probe mixes with RNU6B as endogenous control (Applied Biosystems). The specific reverse transcription of miR-183-5p, miR-96-5p, miR-182-5p, and RNU6B was performed with the TaqMan MicroRNA Reverse Transcription Kit (Applied Biosystems) according to the manufacturer’s protocol modified as follows: the reaction volume was scaled up to 30 µl and 20 ng RNA were used as template. The reverse transcription was carried out as a multiplex reaction, containing the RT-primer for all three miRs and the control in one reaction. The subsequent qPCR reactions were set up according to the manufacturer’s protocol using TaqMan Universal PCR Master Mix II, No UNG (Applied Biosystems) and the miR-specific primer/probe mixes. The runs were performed on a StepOne Plus Real-time PCR system (Applied Biosystems) and the data analyzed using the StepOne Software v2.1 (Applied Biosystems). Relative expression against RNU6B (fold change) was calculated using the ΔCt-algorithm.

### Identification of target genes

Potential target genes of the miR-183 cluster for further analyses were selected by consulting online databases miRWalk 2.0 (http://zmf.umm.uni-heidelberg.de/apps/zmf/mirwalk2/) and miRTarBase (http://mirtarbase.mbc.nctu.edu.tw/) and the review by Dambal et al. [23] concerning the miR-183/96/182 cluster.

### Immunohistochemistry

As target-specific primary antibodies, a monoclonal mouse-anti-FOXO1, clone 3B6, dilution 1:200 (Biozol, Hamburg, Germany), and a monoclonal mouse-anti-EGFR, clone 3G143, dilution 1:200 (Zytomed Systems) were used. Slides were processed on an automatic IHC system, AutostainerLink48 (Dako, Hamburg, Germany) according to routine protocols. Expression was assessed by scoring the staining of three representative areas with 1 (negative / weak), 2 (moderate), or 3 (strong). A final score was built of the mean values of the triplicates. Samples were classified as FOXO1-low, medium, and high with final scores of < 1.5, 1.5–2.5, and > 2.5, respectively.

### GBM in vitro model

The GBM cell line HROG33 was established from a patient-derived xenograft with experimentally validated *EGFR* amplification. The *EGFR* amplification status of HROG33 is adjustable by adaptation of culture conditions. It was cultivated in DMEM/Ham’s F12 with 2 mM L-glutamine and B-27 in an incubator (37 °C, 5% CO_2_, 95% relative humidity), either without EGF to maintain *EGFR* amplification or supplemented with 30 ng/ml EGF to reduce *EGFR* amplification to normal levels [29, 30].

For CISH and IHC analyses, cell pellets were fixed and embedded according to following procedure:

Cells were harvested, washed twice with PBS, and fixed immediately by resuspending pellets in 4% buffered formalin (Formafix; Grimm, Torgelow, Germany). Cells were subsequently processed following standard procedures [31] to form a conglomerate and then embedded in paraffin using the automated Excelsior AS system (Thermo Scientific, Dreieich, Germany).

For miR expression analysis, freshly harvested pellets were used, and miR extraction and miR-specific qPCR were performed as described above.

For mRNA expression analysis, freshly harvested pellets were used, RNA was extracted using the RNeasy Mini Kit (Qiagen) according to the manufacturer’s protocol. cDNA synthesis was performed using SuperScript II Reverse Transcriptase (Invitrogen, Thermo Fisher). Quantitative PCR was performed using the SensiFast Probe Kit (Bioline, Luckenwalde, Germany) and Primer/Probe Sets for *EGFR*, *EGFRvIII* (both TIB Molbiol, Berlin, Germany), *FOXO1*, and *TBP* (both AppliedBiosystems, Darmstadt, Germany) on an ABI StepOne Plus System (Applied Biosystems) according to the manufacturers’ instructions.

### Statistics and box plots

Statistical analyses for the significance of Nanostring array results were embedded within the nSolver Software and p values provided in the results table. Further statistical analysis for expression values obtained by qPCR or IHC, Wilcoxon tests were applied using RStudio v1.2.5019 (RStudio PBC, Boston, USA). Boxplots were generated with the help of the online tool BoxPlotR (URL: http://shiny.chemgrid.org/boxplotr/). Statistical analyses involving patients’ characteristics and survival analyses were performed using SPSS Statistics, version 28 (IBM, Ehningen, Germany).

## Results

### Classification of GBM tumors by *EGFR* amplification status

Initially, for the miR-array analysis, tumor samples from 24 GBM patients were available (Median age 62.2 years, 12 male and 12 female, IDH1/2 mutation in 1/24 patients).

**Fig. 1:**
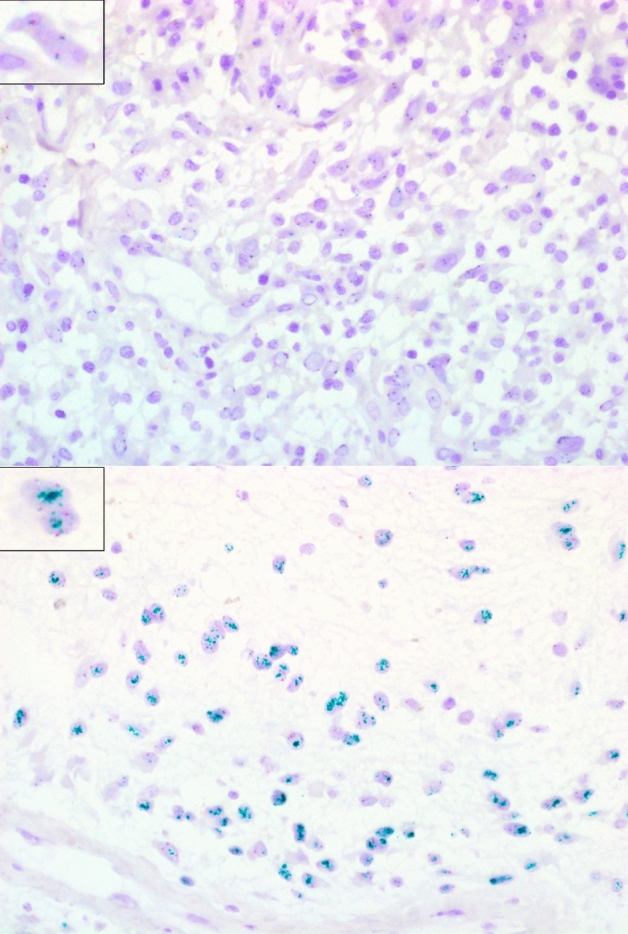
Examples for CISH analysis of glioblastomas with normal *EGFR* (top) and *EGFR* amplification (bottom). Red dots represent centromeres of chromosome 7, green dots are specific for *EGFR*. ×400 magnification. Inlays show, digitally zoomed, single cells representing *EGFR*-normal and *EGFR*-amplified status

These samples were analyzed for *EGFR* amplification by *EGFR*-specific 2C CISH. In 13 samples’ clusters of green signals were observed indicating *EGFR* amplification (*EGFR*-amp), whereas an even distribution of green and red signals was seen in the remaining 11 samples, indicating *EGFR*-normal status (EGFR-*norm*; Fig. 1, Supplementary Fig. 1).

For further analyses, samples from another 56 patients were obtained (median age 66.4 years, 32 male and 24 female; IDH status unknown), whereof 20 showed *EGFR* amplification and 36 had an *EGFR*-normal status (Supplementary Table 1). None of the samples showed an aneuploidy for chromosome 7.

Considering patients’ characteristics like sex and age of diagnosis, none of them showed significant correlations with EGFR amplification state.

### miR screening revealed upregulation of the miR-183/96/182 cluster in *EGFR*-amp GBMs

The group-wise comparison of the miR expression profiles obtained by the Nanostring nCounter assays revealed only moderate changes in expression between the *EGFR-*amp and *EGFR*-normal tumors. The differential expression of miRs downregulated in *EGFR*-amp barely exceeded a factor of 3.5× (Supplementary Table 1) and were, therefore, not considered further in this study. Similarly, most miRs upregulated in *EGFR*-amp tumors did not show pronounced changes in expression levels compared to *EGFR*-norm group with factors up to 3.2× (Supplementary Table 2). Only three miRs—miR-182-5p, miR-96-5p, and miR-183-5p—clearly and significantly outperformed this expression range with upregulation factors of 10.7× (*p* = 0.0015), 6.9× (*p* = 0.0032), and 6.4× (*p* = 0.0019), respectively (Supplementary Table 2).

**Fig. 2:**
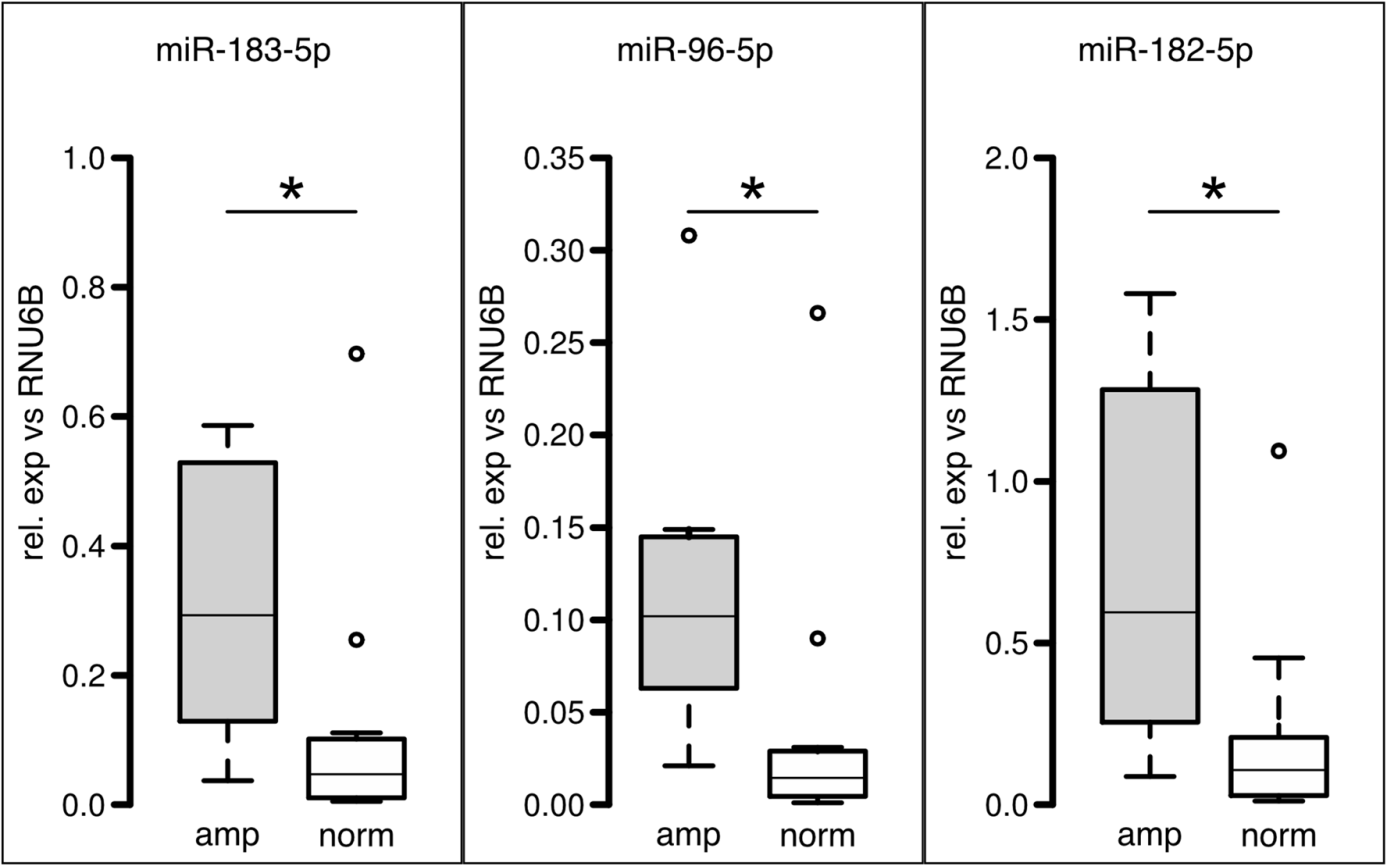
Verification of miR-array data for the initial 24 cases. Boxplot representation of miR-specific qPCR for *EGFR*-amplified samples (gray) and *EGFR*-normal samples (white) showing relative expression normalized against endogenous control RNU6B. Bars represent median values, boxes contain 50% of data points for each group, whiskers extend 1.5 times the interquartile range from the 25th and 75th percentiles, empty circles: outliers. Asterisks indicate significance (*p* < 0.05)

Verification of these array data was performed by quantitative PCR of mature miRs 182-5p, 96-5p, and 183-5p and confirmed their significant upregulation in *EGFR*-amp tumors showing a median overexpression of 5.56× (*p* = 0.003), 7.29× (*p* = 0.003), and 6.23× (*p* = 0.006), respectively (Fig. 2).

To evaluate the significance of the co-occurrence of *EGFR* amplification and miR-183/96/182 cluster expression, the analysis was extended to a total of 80 cases (including the samples used for initial array screening).

**Fig. 3:**
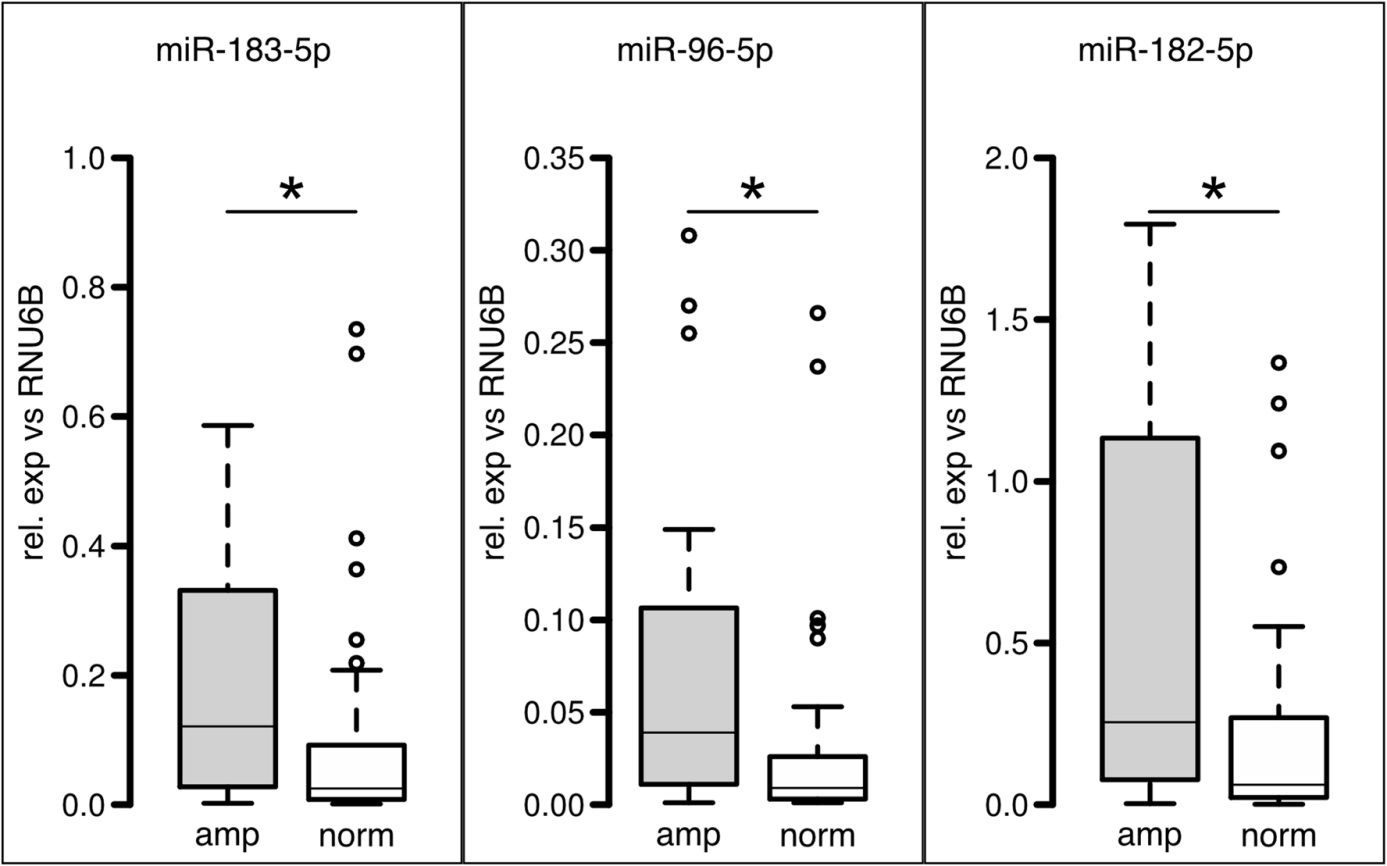
Expression analysis by miR-specific qPCR for all 80 cases. Boxplot representation of miR-specific qPCR for *EGFR*-amplified samples (gray) and *EGFR*-normal samples (white) showing relative expression normalized against endogenous control RNU6B. Bars represent median values, boxes contain 50% of data points for each group, whiskers extend 1.5 times the interquartile range from the 25th and 75th percentiles, empty circles: outliers. Asterisks indicate significance (*p* < 0.05)

The miR-specific qPCR analyses revealed again significant higher expression of miR-182-5p, miR-96-5p, and miR-183-5p showing median fold changes of 4.33× (*p* = 0.003), 4× (*p* = 0.003), and 4× (*p* = 0.006), respectively (Fig. 3). On the miR cluster expression, neither patients’ age at diagnosis nor gender had a significant influence.

### Expression of miR-183/96/182 cluster target FOXO1 is decreased in *EGFR*-amp tumors

Combined search of miR-target databases and literature (based on the review of Dambal et al. [23]) revealed *FOXO1* as the most prominent target of all three members of the miR-183/96/182 cluster.

FOXO1 immunohistochemistry was successful in 74 cases. Expression level scoring classified 31 (40.8%) samples as FOXO1-low, 33 (43.4%) samples as FOXO1-medium, and 12 (15.8%) samples as FOXO1-high.

**Fig. 4:**
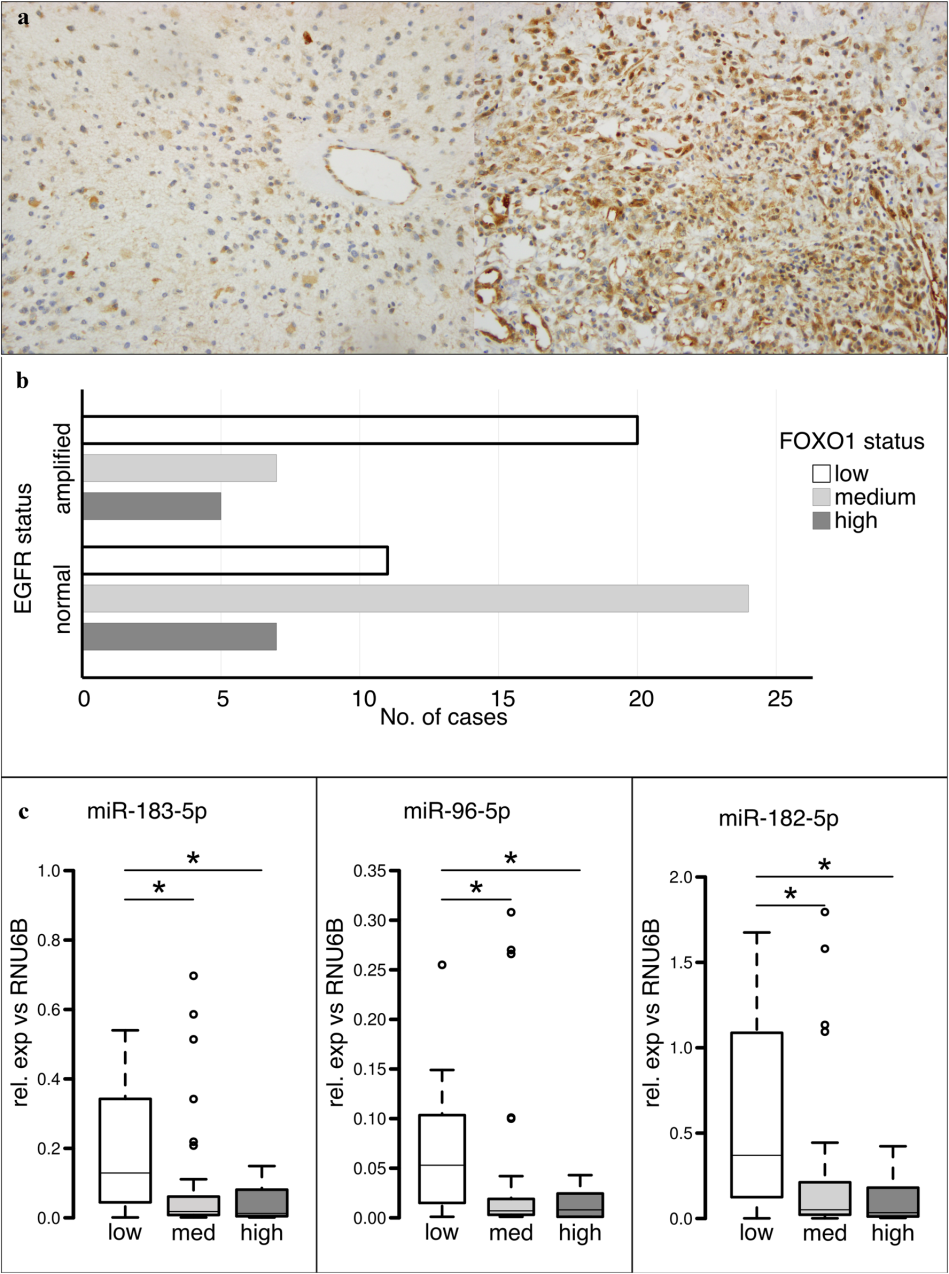
Expression analysis of miR-183/96/182 cluster target FOXO1. **a** Examples for FOXO1 IHC of glioblastomas with *EGFR* amplification (left) and normal *EGFR* (right) showing weak and strong FOXO1 staining, respectively. ×200 magnification. **b** Bar chart showing number of *EGFR*-amplified (top) and *EGFR*-normal (bottom) cases for each FOXO1 expression status (white = low; light gray = medium; and dark gray = high) **(c)** boxplot representation of miR-specific qPCR for samples with low (white), medium (light gray), and high (dark gray) FOXO1 expression, showing relative expression normalized against control RNU6B. Bars represent median values, boxes contain 50% of data points for each group, whiskers extend 1.5 times the interquartile range for the 25th and 75th percentiles, empty circles: outliers. Asterisks indicate significance (*p* < 0.05)

*EGFR*-amplified tumors showed a significant (*p* = 0.004) lower expression of FOXO1 then *EGFR*-normal samples: in *EGFR*-normal samples, 11/42 (26.2%) showed low, 24/42 (57.1%) showed medium, and 7/42 (16.7%) showed high FOXO1 expression. In *EGFR*-amplified samples, 20/32 (62.5%) showed low, 7/32 (21.9%) showed medium, and 5/32 (15.6%) showed high expression. (Fig. 4a, b).

Correlation analysis of FOXO1 expression and miR expression revealed a significant higher expression of miR-183-5p, miR-96-5p, and miR-182-5p in FOXO1-low samples than in FOXO1-med and FOXO1-high samples (Fig. 4c).

miR-183-5p showed in FOXO1-low samples a medium fold change of 6.5× compared to FOXO1-medium samples (*p* = 0.0009), and of 13× compared to FOXO1-high samples (*p* = 0.0045).

miR-96-5p showed in FOXO1-low samples a medium fold change of 5× compared FOXO1-medium samples (*p* = 0.0021) and of 5× compared to FOXO1-high samples (*p* = 0.0037).

miR-182-5p showed in FOXO1-low samples a median fold change of 7.4× compared to FOXO1-medium samples (*p* = 0.0004) and 12.33× compared to FOXO1-high samples (*p* = 0.0013).

Neither patients’ age at diagnosis nor gender had a significant influence to FOXO1 expression.

### Inverse correlation of EGFR and FOXO1 expression in vitro

**Fig. 5:**
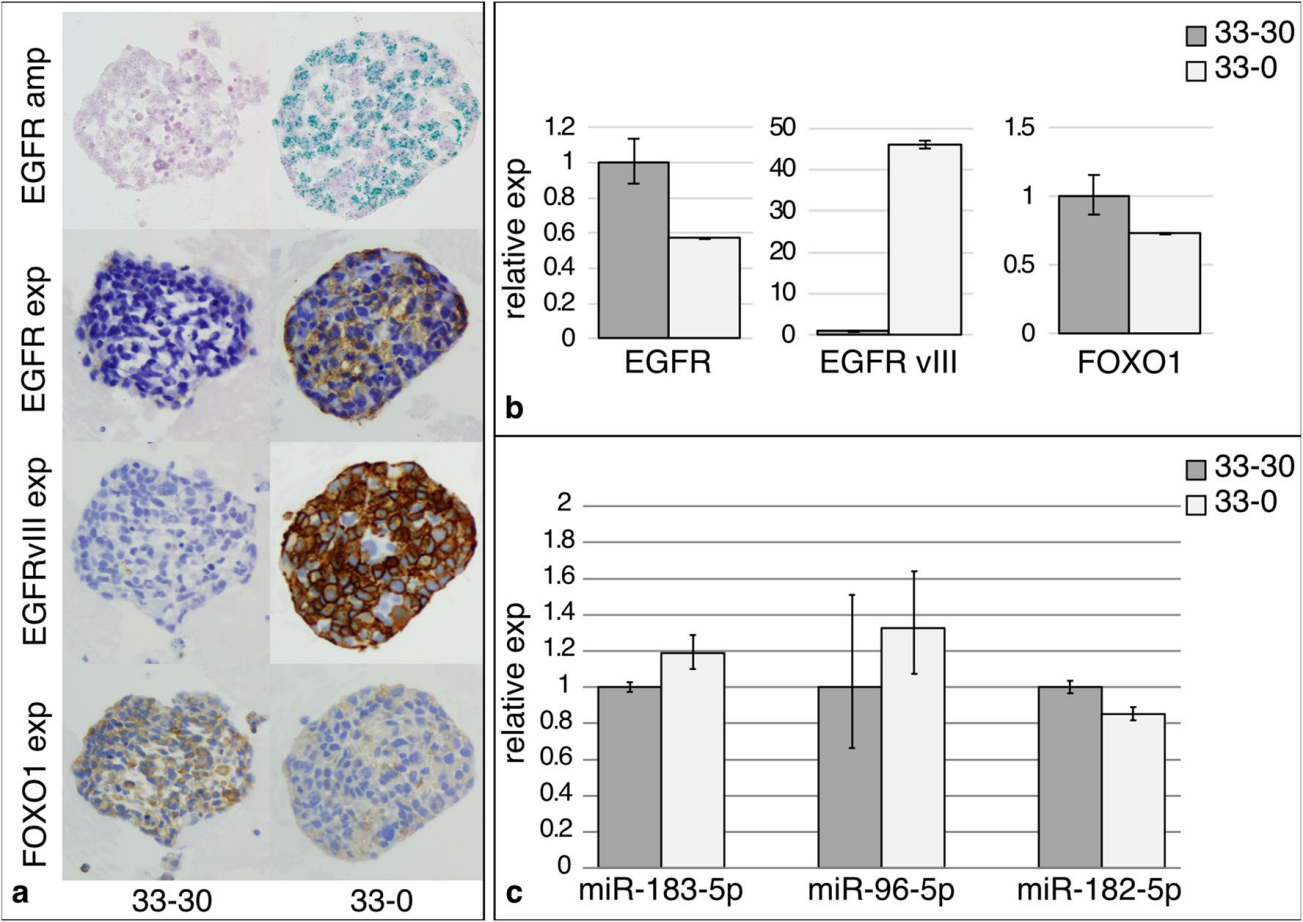
**a** Analysis of HROG33 cells grown with 30 ng (left, 33–30) and without (right, 33–0) EGF Representative pictures showing, from top to bottom, *EGFR* amplification by CISH analysis; EGFR expression, EGFRvIII expression, and FOXO1 expression by IHC. Magnification ×400. **b** Relative quantification of *EGFR* expression, *EGFRvIII* expression, and *FOXO1* expression by qPCR. **c** Relative expression analysis of miRs 183-5p, 96-5p, and 182-5p by miR-specific qPCR. Expression values of 33–30 cells are used as calibrators and set to 1

Cultured HROG33 cells, either cultured under standard condition with 30 ng EGF/ml medium (termed “33–30”) or without EGF supplement (termed “33–0”), were comparatively analyzed with regard to *EGFR* amplification and expression of EGFR, EGFRvIII, and FOXO1. 33–30 cells lost their *EGFR* amplification, whereas 33–0 cells retained the amplified status, as shown by CISH in Fig. 5a, top row. For EGFR and EGFRvIII, a stronger protein expression, analyzed by IHC, was seen in 33–0 cells, EGFRvIII showing an even stronger staining than EGFR (Fig. 5a, second and third row). For FOXO1, an inverse correlation with *EGFR* amplification and EGFR and EGFRvIII expression was observable, as 33–0 cells show a weaker stain in FOXO1 IHC.

Interestingly, looking at the mRNA level, the expression of *EGFR* (wt) is in 33–0 cells surprisingly weaker than in 33–30 cells (Fig. 5b, left), indicating a post-transcriptional control of EGFR expression. For *EGFRvIII*, the increase of expression in 33–0 cells is immense, which reflects the IHC data, a fold change of more than 45× compared to 33–30 cells (Fig. 5b, middle). *FOXO1* shows on the mRNA level a slight decrease of expression in 33–0 cells compared to 33–30 cells (Fig. 5b, right). This is concordant with the IHC data, although the effect on the protein level seems to be stronger, also indicating possible post-transcriptional regulatory mechanisms. The expression levels of miR-183-5p, miR-96-5p, and miR-182-5p remain virtually unchanged (Fig. 5c), unlike the observed upregulated miR expression in EGFR-amplified primary tumors.

## Discussion

MicroArray-profiling analysis revealed upregulation of the miR-183/96/182 cluster in *EGFR*-amplified glioblastoma, comprising the mature miRs 183-5p, 96-5p, and 182-5p. Regarding individual miR expression values, these partly overlapped between the groups, *EGFR*-amp and *EGFR*-norm. Nevertheless, in the group-wise comparison, the upregulation of these three miRs was significant. The initially mentioned *EGFR* amplification-dependent upregulated miR-9 [21] showed in our study a fold change of 1.99, whereas miR-200c, significant target in the study of Serna [22] revealed a fold change of − 2.59, both not reaching top level, yet significant, deregulation (Suppl. Table 1). It must be mentioned that for miR-200 (a, b, and c), only the mature -3p miRs are included in the assay.

The three members of the miR cluster are considered to play multiple roles in several types of cancer, influencing many cellular functions and targeting a multitude of mRNAs, partly in common, partly unique for each of these miRs [23].

In glioblastoma and other gliomas, the miR-183/96/182 cluster is mostly considered to be oncogenic. MiR-183 expression denotes a worse prognosis [32], increased proliferation, and invasion by inhibiting *NEFL* leading to upregulation of *mTOR* [25] and promoting angiogenesis by *HIF1A* upregulation via *IDH2* repression [33]. Although not much is known about the role of miR-96 in glioblastoma, it is associated with oncogenic functions by influencing the Wnt-pathway, combined with worse prognosis [34] and enhanced angiogenesis [35]. Nevertheless, in some other cancers, e.g., nasopharyngeal or pancreatic cancer, it is considered to have tumor suppressor functions targeting MTAI, thus, inhibiting proliferation and invasion [36, 37].

The role of miR-182 in glioblastoma is regarded as controversial. In several studies, miR-182 expression is correlated with prolonged survival [38] and enhanced sensitivity to chemotherapy and may oppose tumorigenesis [39]. Additionally, pro-apoptotic effects have been described [40, 41], and this miR downregulates growth and migration of GBM cells [39, 42]. In contrast, other studies have shown an association of miR-182 expression with a worse prognosis [43, 44] and oncogenic features, like growth promotion [45], sustained *NF-kB* activation [46] and enhanced invasion [47]. miR-182 is described to be activated by TGFb, which in turn can be stimulated by EGFR/EGFRvIII signaling via miR-524 silencing [48]. However, our Nanostring based miR-profiling showed no differential expression of miR-524.

Furthermore, it is shown that the knockdown of the whole cluster increases apoptosis and sensitivity to temozolomide in glioblastoma [24]. Also in other tumor entities, the miR-183/96/182 cluster is widely considered to be oncogenic [23].

*FOXO1* is described as a prominent target, of all three members of the miR-183/96/182 cluster [23]. In our study, the expression of FOXO1 was lower in *EGFR*-amplified tumors. Although IHC is not considered a quantitative method, the differences of FOXO1 expression between *EGFR*-amplified and *EGFR*-normal tumors were obvious. In lung cancer, *FOXO1* repression by miR-183 is described as an anti-apoptotic mechanism [26]. *FOXO1* is also considered as a pro-apoptotic factor in GBM [27] and even as a potential target of directed therapy [28].

The reportedly worse prognosis of *EGFR*-amplified tumors [6] is consistent with a mechanistic scenario whereby *EGFR* amplification leads to repression of *FOXO1*, either via the miR-183/96/182 cluster, direct targeting by EGFR and/or downstream factors, or a synergistic effect of both. Loss of its anti-apoptotic activity may render these tumors more aggressive.

The in vitro data obtained in this study contradict the direct influence of EGFR to the miR cluster, at least in the single-cell line model available. Modulation of *EGFR* amplification and expression did not lead to a differential miR-183/96/182 cluster expression, but, identical to the majority of the patients’ samples, the FOXO1 expression was inversely correlated with *EGFR* amplification and expression. This supports other, or additional, miR-183/96/182 cluster-independent effects of EGFR expression to *FOXO1* regulation. In accordance with this it is known that EGFR signaling can promote downregulation of FOXO-genes via the AKT pathway activation leading to inhibitory phosphorylation [49].

Interestingly, for EGFR and FOXO1 expression, the EGFR modulation showed effects rather on the protein level. The changes on the RNA level are rather minor (*FOXO1*) or even contrary (*EGFR*), indicating post-transcriptional effects. For *EGFRvIII*, the high increase of RNA expression is accordant to the observed increased protein expression, maybe rendering EGFRvIII as a principal oncogenic component for this cell line model.

The reason for the lack of influence to the miR cluster expression remains speculative. Maybe other control mechanisms of miR expression predominate in this cell line model as several transcription factors are known to play a role in regulating the whole cluster or single miRs thereof (reviewed in [23]). In contrast to primary tumors analyzed, the in vitro model lacks a tumor microenvironment, which could also have an effect to differential miR expression. Not all the primary tumors with *EGFR* amplification show miR cluster upregulation and FOXO1 downregulation, demonstrating heterogeneity. Thus, the results of only a single-cell line model, especially in consideration of this heterogeneity, are not eligible to draw common conclusions. Therefore, intensifying studies with more models, maybe in vivo PDX tumors with a microenvironment, and functional assays with overexpression as well as silencing of miR-183-5p, miR-96-5p, and miR-182-5p would be necessary in the future to give more inside into these complex mechanisms.

Despite yet lacking evidence for a functional relationship, we have shown that there exist significant correlations between *EGFR* amplification, upregulation of the miR-183/96/182 cluster and FOXO1 downregulation, rendering this miR cluster an interesting subject for further analysis concerning its influence on tumorigenicity and its potential as target for directed therapy by silencing approaches.

In summary, this study shows an upregulation of the miR-183/96/182 cluster in *EGFR*-amplified glioblastoma, accompanied by reduced expression of FOXO1. Although the functional context remains to be identified, these data provide a solid molecular basis for further functional in vitro or in vivo assays to analyze the effects of miR-183/96/182 cluster and FOXO1 deregulation on proliferation, tumorigenicity, or chemoresistance, and to investigate whether they offer potential targets for new directed therapy approaches.

## Supplementary Information

Below is the link to the electronic supplementary material.

Supplementary file1 (JPG 4760 kb) **Fig.1** *EGFR* specific CISH analysis of the 24 glioblastoma tissues used for miR-screening. Red dots represent centromeres of chromosome 7, green dots are specific for EGFR. #1 - #13 show EGFR amplified tumors, #14 - #24 EGFR normal tumors. ×400 magnification

Supplementary file2 (XLSX 10 kb) **Table 1** Patiens' data showing their gender, age at dignosis and *EGFR* amplification status

Supplementary file3 (XLSX 31 kb) **Table 2** Results of Nanostring Human v3 miRNA assays showing fold changes and their *p* values of EGFR amplified vs EGFR normal glioblastomas

## Data Availability

Raw data and images can be obtained from the authors.
