# Supplementary figures and images for: The miR-183/96/182 cluster is upregulated in glioblastoma carrying EGFR amplification
Source: Mol Cell Biochem. 2022 Apr 29;477(9):2297–307. doi: 10.1007/s11010-022-04435-y (PMC9395473; doi:10.1007/s11010-022-04435-y)

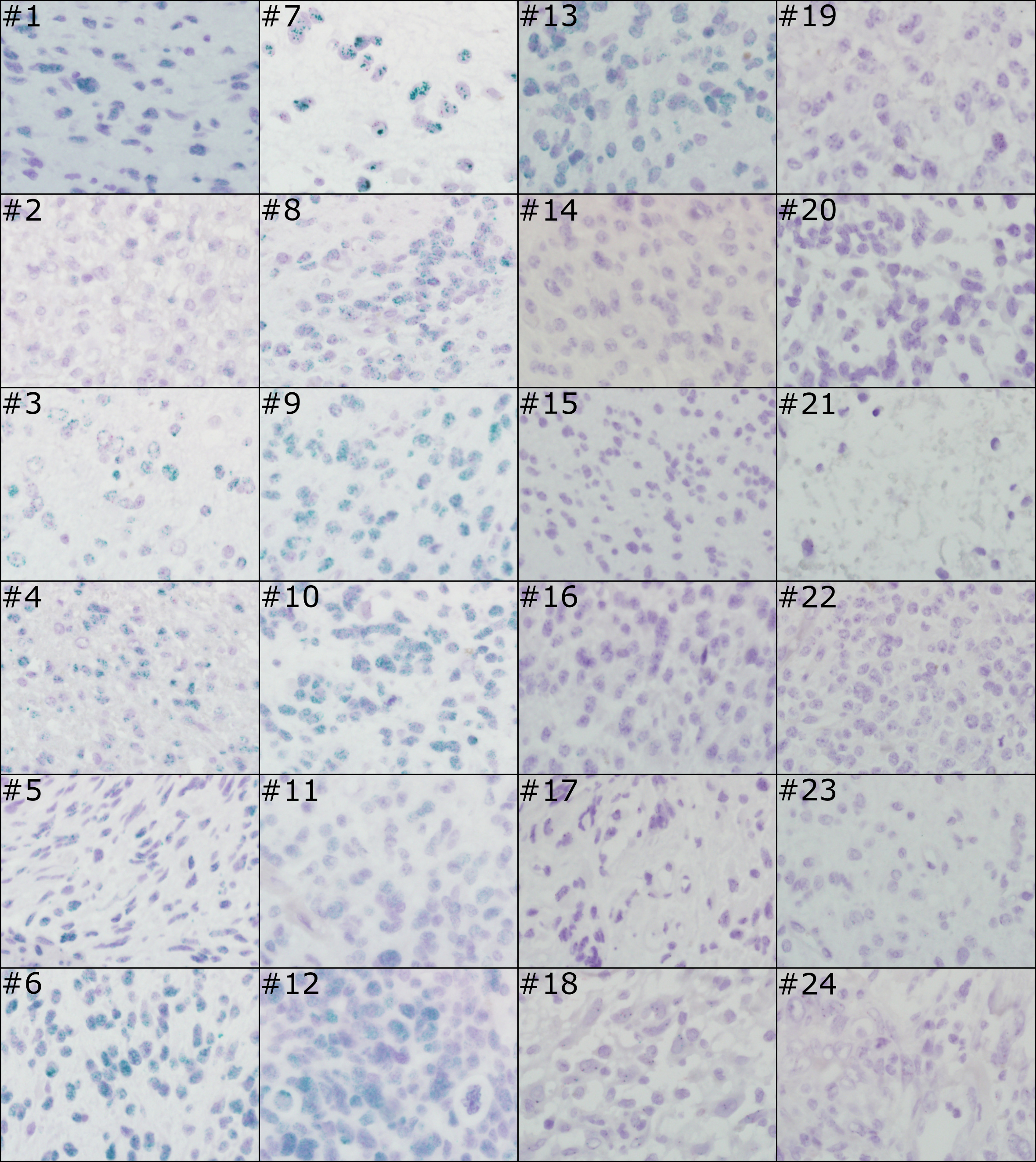

Supplement: Supplementary file 1 — Supplementary file1 (JPG 4760 kb) Fig.1 EGFR specific CISH analysis of the 24 glioblastoma tissues used for miR-screening. Red dots represent centromeres of chromosome 7, green dots are specific for EGFR. #1 - #13 show EGFR amplified tumors, #14 - #24 EGFR normal tumors. ×400 magnification [file 11010_2022_4435_MOESM1_ESM.jpg]
